# Innovative numerical modeling for predicting soil relaxation in the design of twin circular culverts

**DOI:** 10.1038/s41598-024-58507-8

**Published:** 2024-04-02

**Authors:** Jim Shiau, Tan Nguyen, Mathew Sams, Paramita Bhattacharya

**Affiliations:** 1 School of Engineering, University of Southern Queensland, Brisbane, QLD Australia; 2 Smart Computing in Civil Engineering Research Group, Ton Duc Thang University, Ho Chi Minh City, Vietnam; 3 Faculty of Civil Engineering, Ton Duc Thang University, Ho Chi Minh City, Vietnam; 4 School of Engineering, University of Southern Queensland, Brisbane, QLD Australia; 5 Faculty of Civil Engineering and Architecture, Indian Institute of Technology Kharagpur, Kharagpur, India

**Keywords:** Twin culverts, Stability, Settlement, Pressure relaxation, Finite difference, FLAC, Civil engineering, Computational science

## Abstract

This study presents a finite difference model for analyzing ground stability and settlement of twin circular culverts in undrained clay. The model is verified through simulations of soil movement and relaxation around a tunnel-boring machine's shield. Stability numbers and ground settlement are evaluated across various culvert geometries and soil ratios and compared to rigorous solutions and previous models. The settlement data obtained is used to determine inflection point parameters for practical culvert design, considering dimensionless ratios. The findings highlight the importance of precise design methodologies that consider soil properties and geometry. The finite difference model proves to be a valuable tool in culvert design, providing accurate analysis of stability and settlement characteristics. The presented design figures and regression equations serve as practical tools for engineers in designing stable twin circular culverts in undrained clay. The study emphasizes the need to carefully consider soil properties and geometry for successful culvert design. In conclusion, the finite difference model offers insights into ground stability and settlement of twin circular culverts. The presented design figures and regression equations support engineers in making informed design decisions, ensuring the stability and long-term performance of culverts in undrained clay conditions.

## Introduction

The demand for culverts and underground infrastructure has grown significantly in recent years. Due to the limited scope for above-ground modifications and the abundance of unused underground space, culverts have become a popular choice for public transportation and water supply infrastructure. With the advancement of tunnel boring machines (TBMs), culverts can now be constructed in increasingly challenging ground conditions, including very soft soils. Consequently, geotechnical engineers bear greater responsibility for culvert design in these conditions, where soil mechanics play a critical role.

In culvert design, the stability and settlement of the ground are critical considerations for geotechnical engineers. This involves analyzing three key design factors: stabilizing internal pressure (*σ*_*t*_), destabilizing surcharge (*σ*_*s*_), and self-weight (*γH*) pressures, along with the undrained shear strength of the soil (*S*_*u*_). The interaction of these factors can result in either active failure (collapse) or passive failure (blowout) of the culvert system.

To simplify the design process, Broms and Bennermark^1^ introduced the concept of the critical stability number *N*, which combines these factors into a single dimensionless parameter. While Broms and Bennermark's^1^ research primarily focused on the plastic flow of clay soil in vertical retaining wall openings, a similar soil behavior was observed when the difference between *σ*_*t*_ or *σ*_*s*_ remained constant, regardless of their specific values [as expressed in Eq. (1)]. By utilizing the critical stability number *N*, engineers can streamline the design process by considering the combined effect of these design factors. This approach allows for a convenient and efficient assessment of culvert stability, aiding in the development of robust and reliable designs for various geotechnical applications.

1 $$N = \frac{{\sigma_{s} + \gamma H - \sigma_{t} }}{{s_{u} }}$$

where *σ*_*s*_ and *σ*_*t*_ are the surcharge pressure and the internal culvert pressure respectively; *γ* is the soil unit weight, and *S*_*u*_ is the undrained shear strength of the soil, *H* is the depth of the center of the culvert.

Extensive research has been conducted by various scholars to study the stability of culverts. Peck^2^ conducted a comprehensive investigation on culvert construction and deep excavations, while Atkinson and Cairncross^3^ focused on understanding the collapse mechanism. Clough and Schmidt^4^ explored soil behavior and factors influencing stability, and Seneviratne^5^ examined the effects of pore pressure. Mair^6^ utilized centrifugal modeling to experimentally analyze culverts on soft ground. In a separate study, Davis et al.^7^ explored upper and lower bound solutions, considering the pressure ratio (*σ*_*s*_ − *σ*_*t*_)/*S*_*u*_ along with independent parameters such as the depth ratio *C*/*D* and the shear strength ratio *γD*/*S*_*u*_.

These studies have paved the way for the development of computational methods to investigate culvert stability. Notably, recent works by Shiau and Al-Asadi^8–10^, Shiau and Keawsawasvong^11^, and Shiau et al.^12^ have contributed significantly to this field. These computational approaches provide valuable insights and analysis techniques for assessing culvert stability under various conditions. By building upon the knowledge gained from previous physical model studies, these computational methods enhance our understanding of culvert behavior and assist engineers in designing stable and reliable culvert structures.

As the demand for underground infrastructure grows, constructing twin culverts becomes more necessary. However, building twin culverts is more complicated than constructing a single culvert because of the possible interaction between the two. To investigate the stability of twin culverts, researchers have used the stability number, *N*, defined in Eq. (1) as mentioned earlier. Numerous computational methods, such as those employed by Wu and Lee^13^, Osman et al.^14^, and Wilson et al.^15^, have also utilized this approach.

Recently, numerous studies, such as Zheng et al.^16^ and Ağbay and Topal^17^, have explored the mechanical characteristics of twin tunnels in diverse geological conditions. Zheng et al. conducted a large-scale 3D geomechanical model test on closely-spaced twin tunnels, revealing insights into lining mechanics. Ağbay and Topal focused on minimizing surface deformations in inner-city shallow tunnels, introducing a modification factor for settlement prediction.

Other studies, including Do and Wu^18^ and Fang et al.^19^, investigated jointed rock mass behavior and proposed novel analytical approaches for tunnel pressure calculation. Additionally, Jiang et al.^20^ addressed stability using 3D printing models, while Wu et al.^21^ identified the impact of closely-spaced twin tunnels on existing structures. Islam and Iskander^22^ analyzed ground settlement in clay, offering insights for water-rich regions. Furthermore, sophisticated holistic approaches have recently emerged, integrating upper bound limit analysis through discretization method of isogeometric analysis with machine learning techniques, aimed at investigating tunnel stability with unprecedented depth and accuracy^23,24^.    

Moreover, Zheng et al.^16^ examined settlement behavior in twin-tunnel arrangements, clarifying the relationship between soil stiffness changes and settlements. Qu et al.^25^ proposed a machine learning approach for reliability assessment, and Nie et al.^26^ integrated geological methods for water-rich granite identification. Finally, Shen et al.^27^ explored deformation mechanisms in twin tunnels. Xiang et al.^28^ investigate shallow tunnel deformation and failure mechanisms, emphasizing the influence of surrounding material strengths and challenging the applicability of the Peck formula. Zhang et al.^29^ systematically study tunnel deformation during excavation, exploring the impact of ground loss ratio and settlement trough volume. Their findings enhance our understanding of urban tunnel construction effects. Collectively, these studies provide valuable insights for enhancing the safety and efficiency of twin tunnel construction.

It was observed that previous studies primarily focused on soil stability during collapse, with limited research on settlement estimation. To address this research gap, the objective of this study is to develop a numerical model capable of simulating the movement and relaxation of soil surrounding the shield and lining annulus induced by the overcutting and grouting of the culvert void using a tunnel-boring machine (TBM). The model aims to bridge the gap between soil stability and settlement analysis. To assess the stability of the twin culvert, the study utilizes the stability number proposed by Broms and Bennermark^1^ to interpret the obtained results. In terms of settlement analysis, a regression analysis of the settlement profile data is performed using a commonly used Gaussian equation. This regression analysis allows for the determination of the inflection point parameters (*i*_*x*_) for practical ranges of culvert geometry and soil properties. Based on these findings, design charts are developed, and practical examples are presented to demonstrate the application of these design figures. Through this study, the numerical model and design charts provide valuable tools for engineers to analyze both the stability and settlement aspects of twin culverts. The findings contribute to a comprehensive understanding of culvert behavior and assist in the design of reliable and robust culvert structures in practical engineering applications.

## Stability analysis

### Problem definition and stability simulations

**Figure 1:**
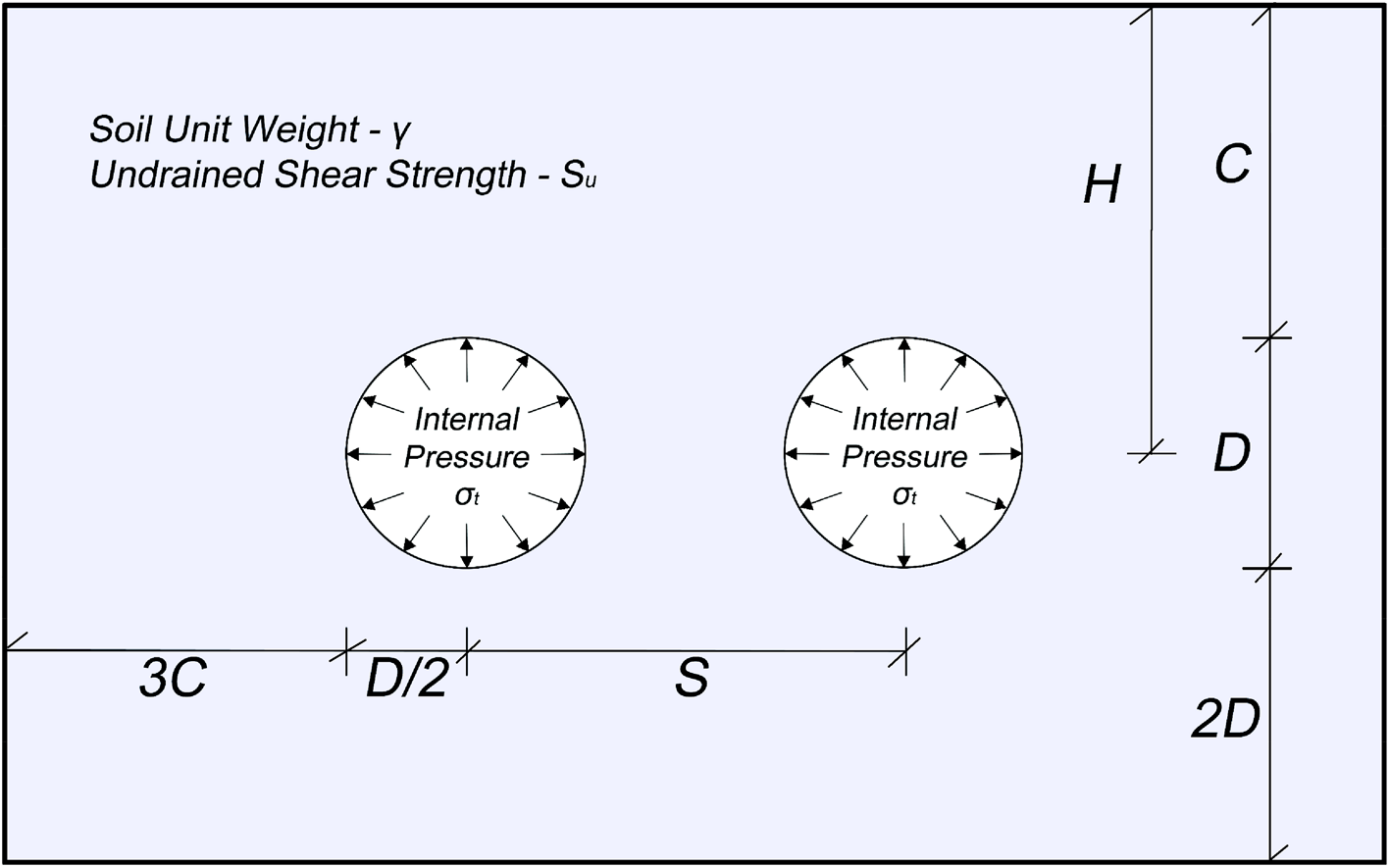
A schematic diagram of the problem.

To simplify the complex three-dimensional problem of culvert construction, a 2D plane strain condition can be assumed by considering the transverse section and assuming a long twin culvert. Figure 1 illustrates a twin culvert with a diameter (*D*) and soil overburden (*C*). The soil body in this study is simulated using a uniform Mohr–Coulomb model, taking into account key parameters such as undrained shear strength (*S*_*u*_) and unit weight (*γ*). This modeling approach allows for a realistic representation of the soil's behavior by considering its shear strength and density. The spacing between the twin culverts is defined as (*S*). While the elastic modulus and Poisson's ratio of the soil are 2 MPa and 0.49, respectively, these agents do not play a significant role in determining soil stability problems, as reported by Keawsawasvong et al.^30^ and Li et al.^31^.

To evaluate the stability of the soil in twin culverts, researchers typically utilize the original stability number (*N*) introduced by Broms and Bennermark in 1967, as indicated in Eq. (1). However, due to the added complexity involved in twin culverts, it is recommended to use the critical pressure ratio (*PR*) instead [refer to Eq. (2)], which excludes the self-weight, diameter, and shear strength effects (*γD*/*S*_u_) from the equation. The new stability number, also known as the critical pressure ratio, is dependent on the depth ratio *C*/*D*, spacing ratio *S*/*D*, and shear strength ratio *γD*/*S*_*u*_, as demonstrated in Eq. (2).

2 $$PR = \frac{{\sigma_{s} - \sigma_{t} }}{{s_{u} }} = f\left( {\frac{C}{D},\frac{\gamma D}{{s_{u} }},\frac{S}{D}} \right)$$

The derived equation enables the generation of practical stability charts that prove valuable in design applications. By employing dimensionless ratios, the results of this study can be extended to different scenarios with diverse physical characteristics, as long as the soil strength ratio, depth ratio, and spacing ratio fall within the specified parameter range. The parameters considered in this investigation encompass a wide range of realistic values, including *γD*/*S*_*u*_ values ranging from 1 to 5, *C*/*D* values ranging from 1 to 5, and *S*/*D* values ranging from 1.5 to 20. This wide coverage ensures that the design charts developed in this study are applicable to a broad range of culvert designs, providing utility and practicality in various engineering scenarios.

**Figure 2:**
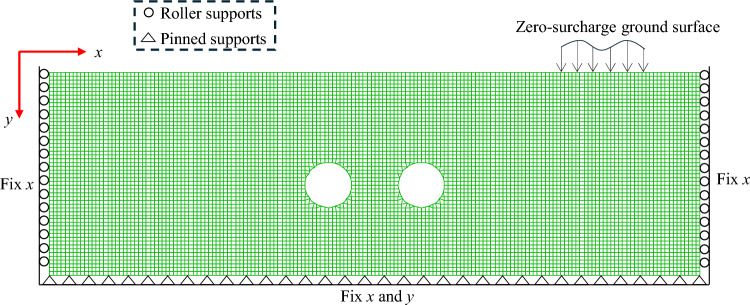
A typical mesh of the unlined twin culverts generated by the developed automatic FISH script.

Figure 2 depicts a representative finite difference mesh employed in this study for solving the twin culvert problem. The boundary conditions showcased in the figure play a crucial role in ensuring an accurate depiction of the entire soil mass. Careful consideration was given to the selection of the soil domain size for each case to ensure that the failure zone of the soil body falls well within the defined domain. The stability analysis of twin culvert problems was conducted using the internal pressure relaxation method, as described by Shiau and Sams^32^. The proposed method systematically decreases the support pressure of the culvert from its initial state until a failure point is identified. At each relaxation step, both the stability number (*N*) and the ground settlement are computed. This iterative process is applied to various culvert geometries and soil ratios, covering a wide range of practical scenarios involving undrained clay conditions.

It is important to acknowledge that in the present study investigating twin culverts, the assumption was made that both culverts experience excavation and relaxation simultaneously and uniformly. This approach, coupled with the adoption of a small relaxation interval, facilitated the accurate determination of the pressure ratio [as expressed in Eq. (2)] leading to collapse. It is important to recognize that the pressure relaxation method used in this study may slightly overestimate the stability number at the collapse point. This is attributed to the discrete step reduction of internal pressure, which deviates from the continuous process. As a result, the internal pressure at the collapse stage might have been relaxed more than required, unless it precisely aligns with the actual collapse stability number. To mitigate this issue, the size of the relaxation intervals was reduced, leading to a significant improvement in accuracy, as demonstrated by Shiau and Sams^32^.

### Discussing stability results

**Figure 3:**
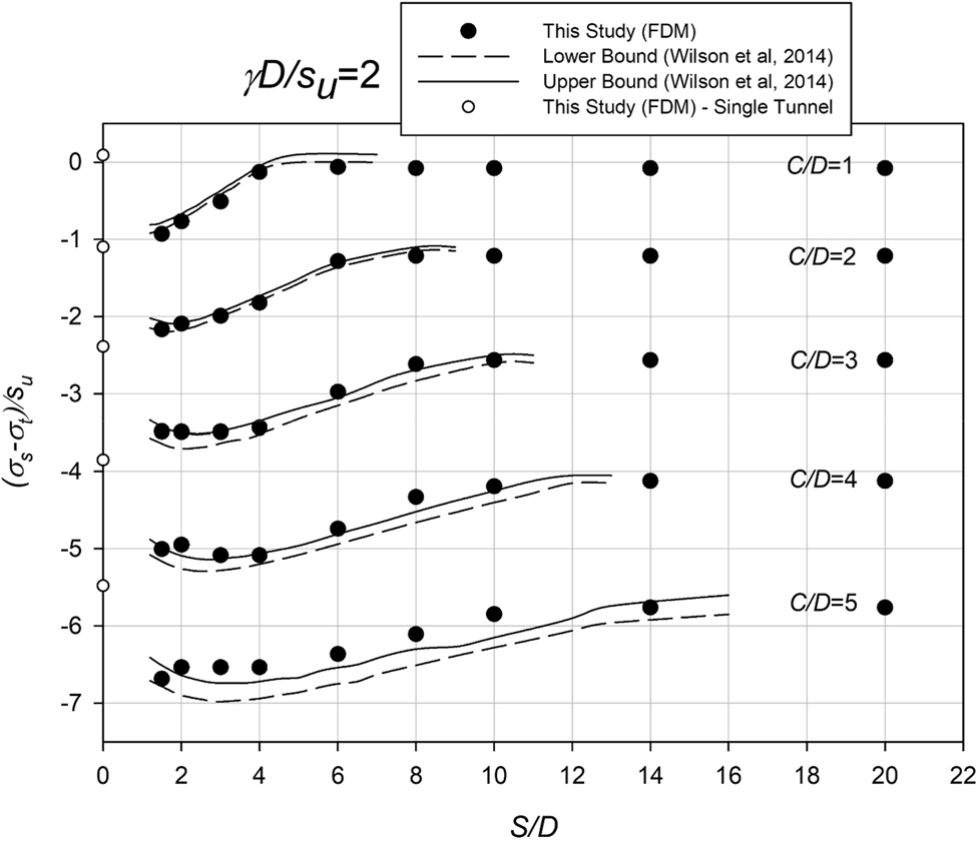
Comparison with other numerical results (*γD/S*_*u*_ = 2).

By utilizing the pressure relaxation method, as described earlier, Fig. 3 presents a comparison between the stability number (*N*) obtained from this study and the upper and lower bounds of Wilson et al.^15^ for a soil strength ratio (*γD*/*S*_*u*_) of 2. The comparison highlights the close agreement between the two approaches. As the distance between the twin Culverts decreases, the required pressure ratio increases proportionally, indicating a larger required *σ*_*t*_. This trend is depicted in Fig. 3, where the pressure ratio eventually reaches a plateau as the spacing between the Culverts increases. Importantly, the depicted figure also includes the critical stability number for a single culvert, represented by a white circular symbol at *S*/*D* = 0. It is intriguing to note that the levelling off the critical stability number for the twin culverts corresponds to that of the single culvert.

In the present study concerning twin culverts, the assumption is made that both culverts undergo excavation and relaxation simultaneously and at an equal rate. When the culverts are positioned in close proximity to each other, with a spacing ratio (*S*/*D*) approaching 1 to 0, the ground settlement profile resembles that of a single culvert. It is worth noting that when the spacing ratio (*S*/*D*) precisely equals 1, the culverts are positioned adjacent to each other, whereas for *S*/*D* < 1, they intersect hypothetically. With a slight increase in the spacing ratio, the interaction effects between the culverts become more noticeable, causing the collapse point to occur at a more negative value of *N*. However, as the spacing ratio continues to increase, the interaction effects diminish, and the behavior of the two culverts resembles that of individual culverts, resulting in a collapse point similar to what is observed in studies focusing on single culverts. These observations are consistent with previous research conducted on this subject.

**Figure 4:**
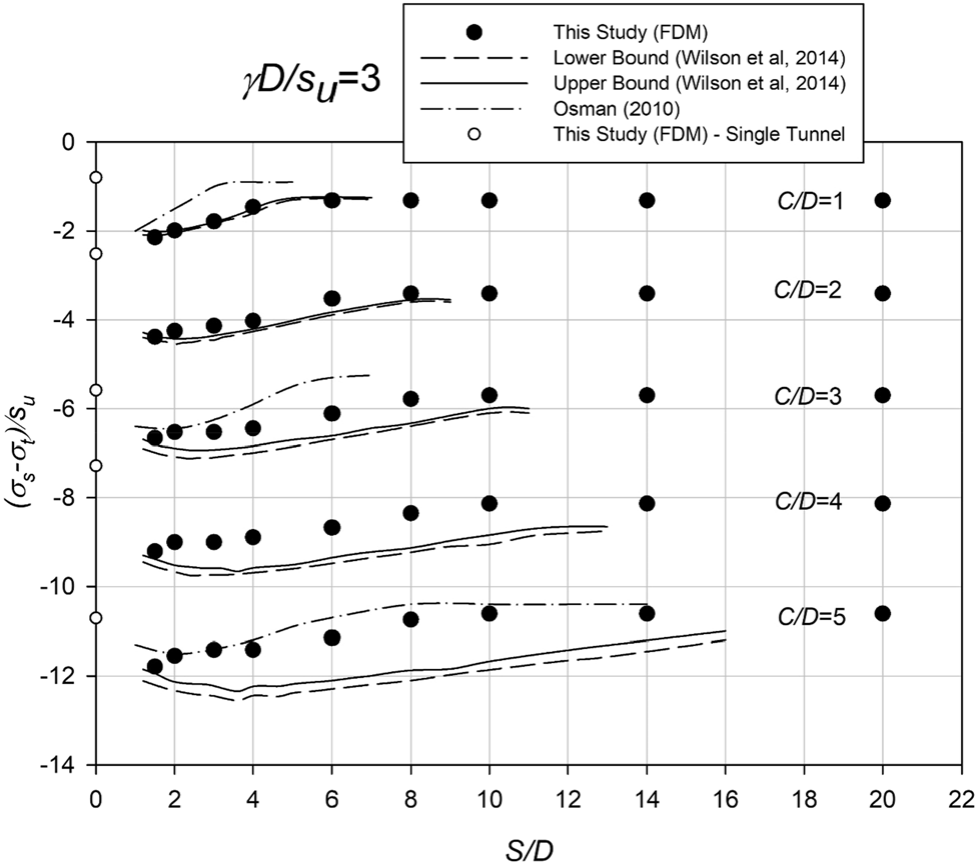
Comparison with other numerical results (*γD/S*_*u*_ = 3).

Figure 4 illustrates a chart resembling Fig. 3; however, it pertains to *γD*/*S*_*u*_ = 3. This chart allows for a comparison between the outcomes obtained in this study, Osman^33^, and the upper and lower solutions from Wilson et al.^15^ using finite element analysis (FELA). Similar to Fig. 3, the critical pressure ratio for a single culvert is represented by a white dot, while the spacing ratio spans from 0 to 20. The comparison reveals a good correspondence between the results of this study and the FELA bounds presented by Wilson et al.^15^ for *C*/*D* = 1. However, the agreement is not as satisfactory for deeper cases (higher *C*/*D*). Conversely, the findings of Osman^33^ align well with the present study's results in shallow cases, but match more closely in deep cases. Consequently, it can be concluded that the results of this study align with the FELA bounds in certain scenarios but deviate in others. Likewise, the outcomes of Osman^33^ exhibit consistency in some cases but not in others.

**Figure 5:**
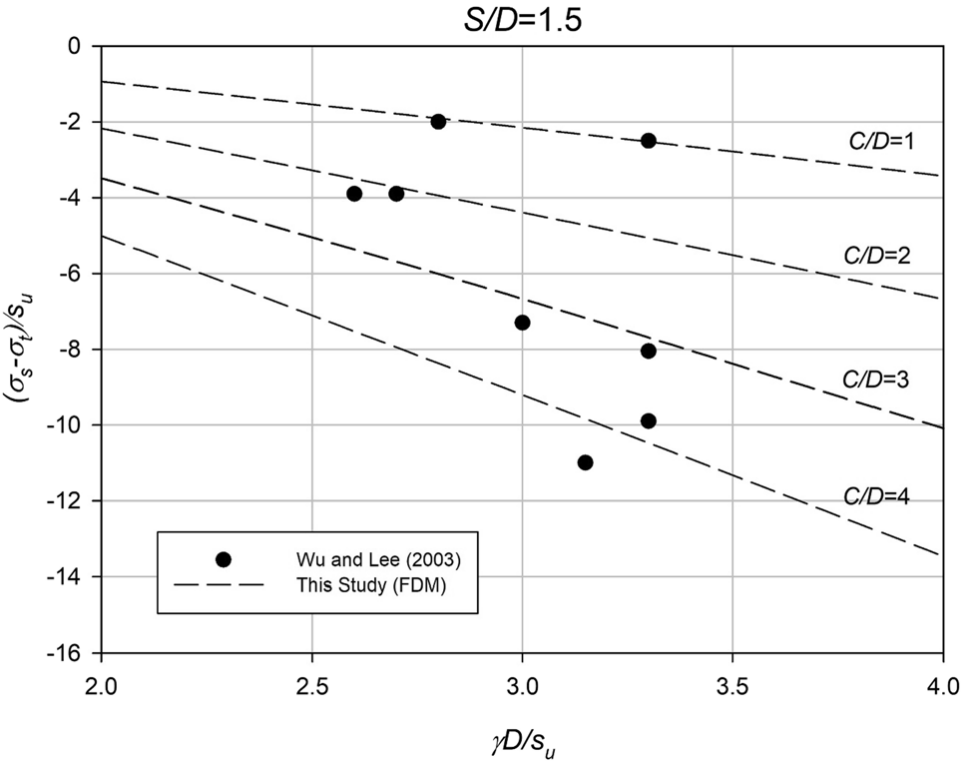
Comparison of results with experimental results (*S/D* = 1.5).

Figure 5 illustrates a comparison between the numerical results of this study and the experimental results of Wu and Lee^13^ for the case of *S*/*D* = 1.5. The experimental results are shown as solid dots, and the numerical results are represented by a dashed line, with two dots for each *C*/*D* value. The comparisons demonstrate good agreement between the two methods, confirming the applicability of the numerical model in producing design charts.

**Figure 6:**
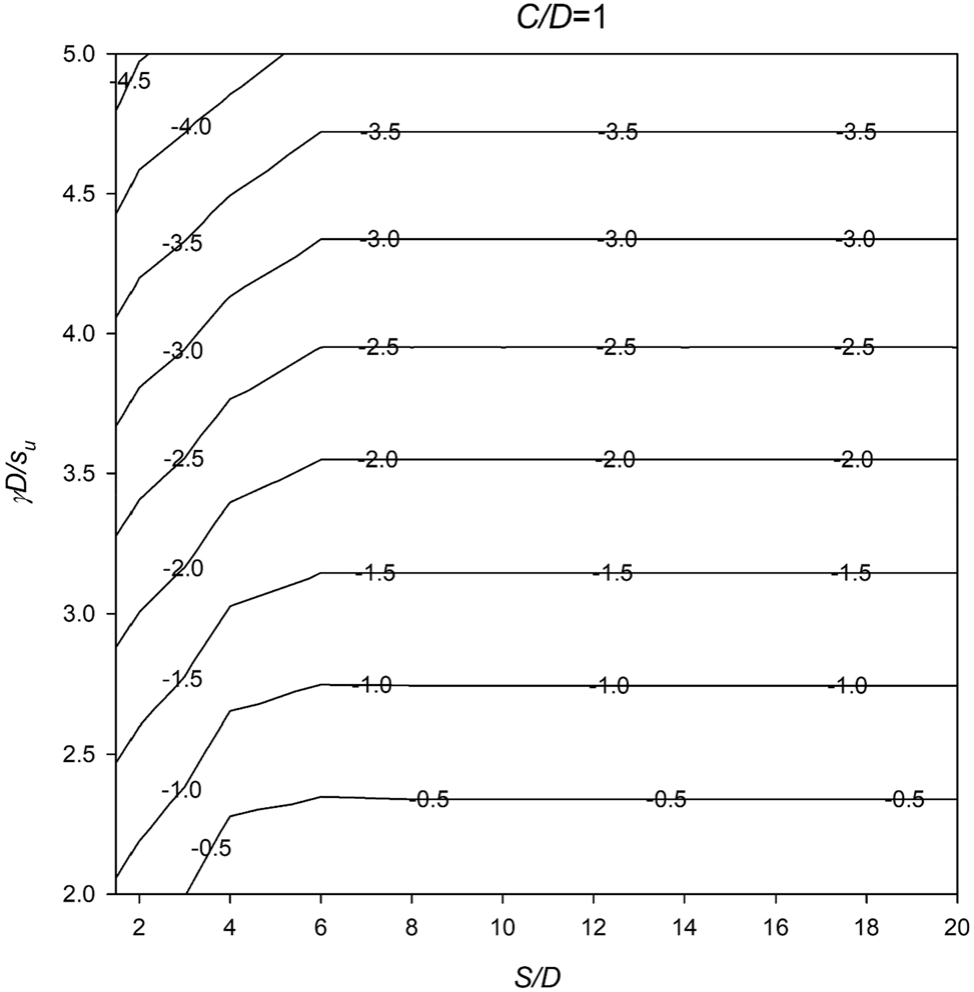
Design chart for the critical pressure ratio *PR* (*C/D* = 1).

**Figure 7:**
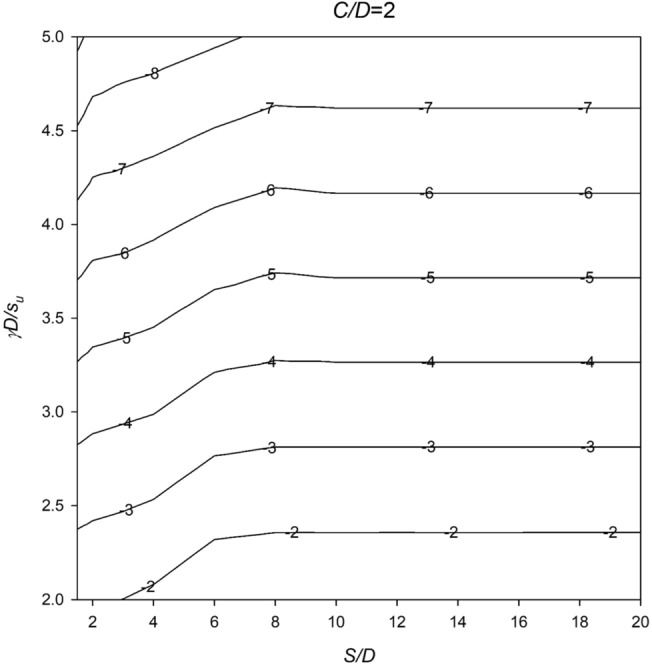
Design chart for the critical pressure ratio *PR* (*C/D* = 2).

**Figure 8:**
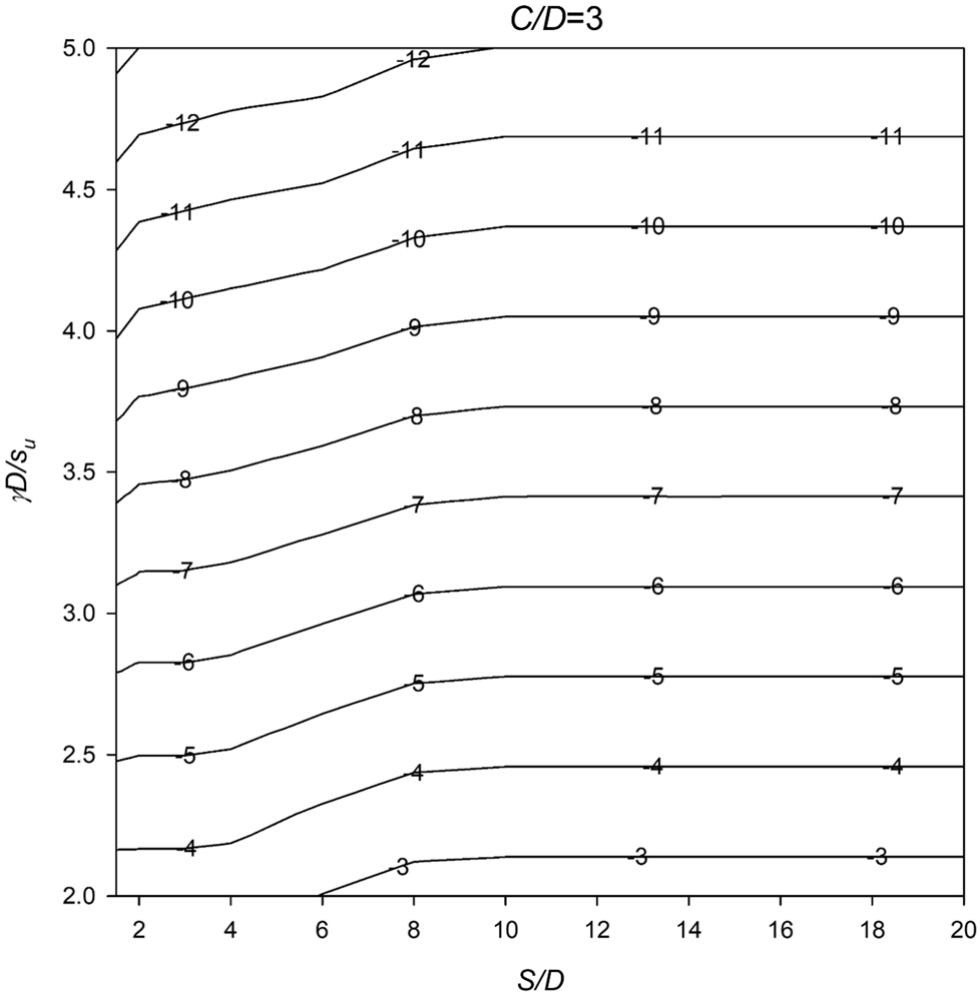
Design chart for the critical pressure ratio *PR* (*C/D* = 3).

**Figure 9:**
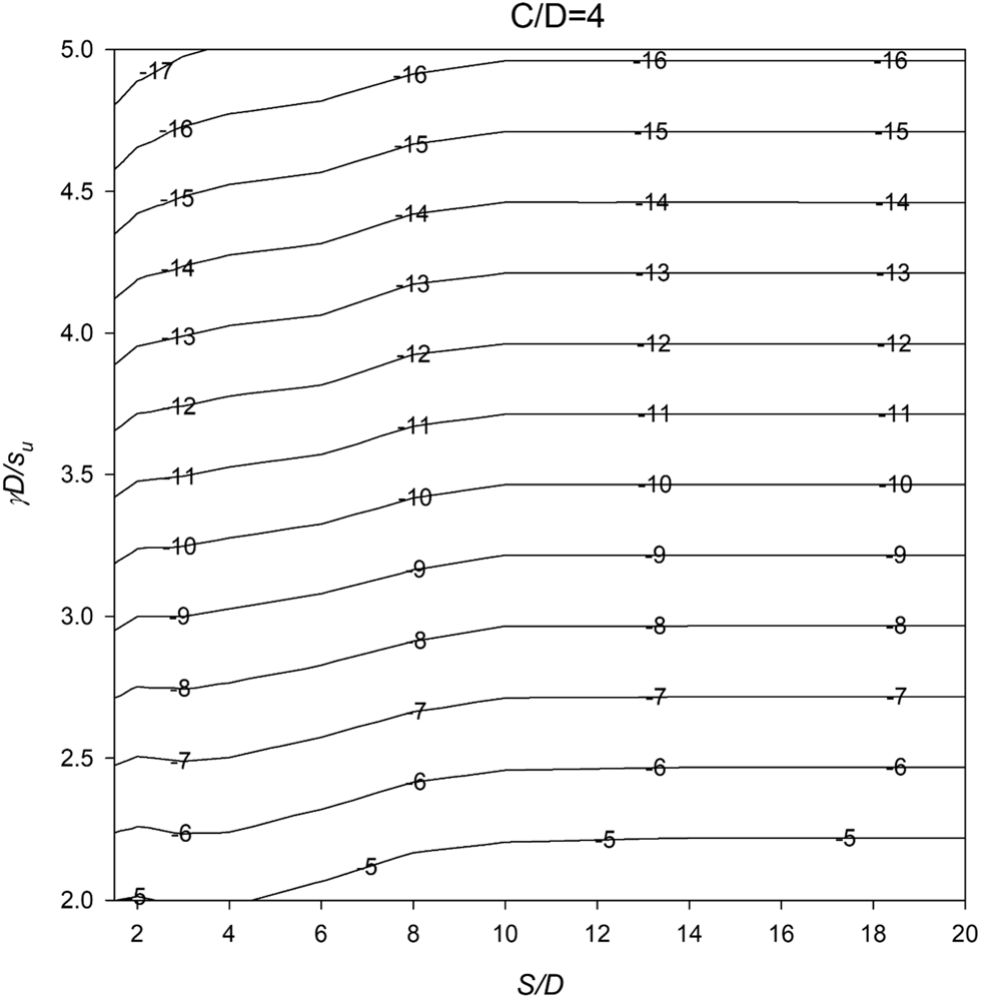
Design chart for the critical pressure ratio *PR* (*C/D* = 4).

**Figure 10:**
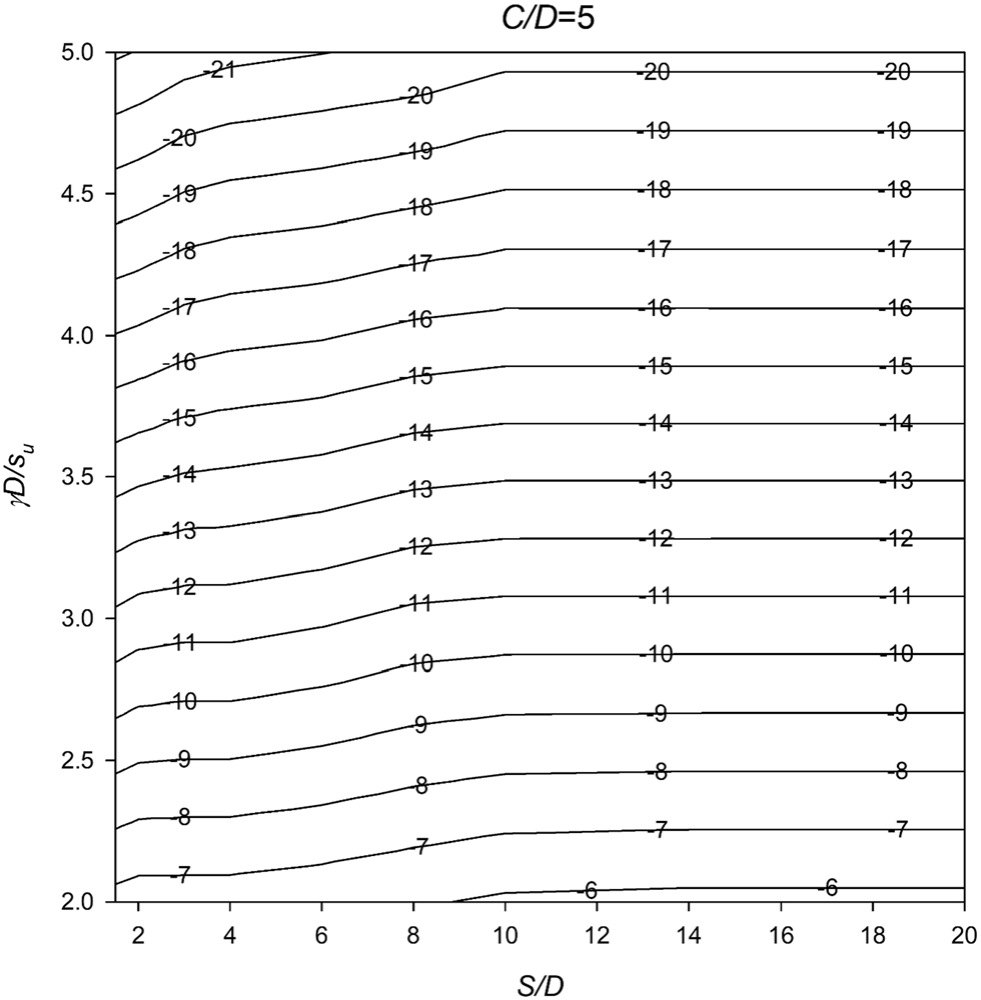
Design chart for the critical pressure ratio *PR* (*C/D* = 5).

In Figs. 6, 7, 8, 9 and 10, a series of meticulously crafted design charts unfolds, each tailored to distinct values of *C*/*D*. These charts provide an intricate exploration of the stability number *N* concerning varied *S*/*D* and *γD*/*S*_u_ values. For instance, Fig. 6, devoted to *C*/*D* = 1, delves into the stability behavior of twin culverts under specific geometric and soil conditions. Progressing through Figs. 7, 8, 9 and 10 with increasing *C*/*D* values, these charts furnish a comprehensive view of *N* variations, ensuring broad applicability across diverse culvert configurations. Serving as indispensable tools, the charts empower geotechnical engineers to navigate the complex interactions between culvert geometry and soil properties.

Within these design charts, engineers discern critical information influencing twin culvert stability. Figure 6 lays the groundwork, exploring stability for *C*/*D* = 1 and shedding light on the effects of different *S*/D and *γD*/*S*_u_ combinations. Figures 7, 8, 9 and 10 progressively expand this exploration, offering nuanced perspectives on how culvert geometry and soil properties changes impact stability. As visual guides, the charts enable practitioners to assess the stability number *N* across diverse scenarios, facilitating informed decision-making in culvert design. By presenting this wealth of information visually, the design charts become essential instruments for geotechnical professionals seeking robust and reliable solutions for twin culvert projects.

The practical utility of these design charts materializes in the subsequent section, where a concrete example illustrates their application without relying on pronouns. This step-by-step illustration demonstrates how engineers can interpret and leverage the information embedded in the charts, underscoring their pivotal role in the engineering decision-making process. The integration of these design charts into the broader framework of the study enhances their significance, transforming them into powerful tools contributing to a comprehensive understanding of twin culvert stability under varying real-world conditions.

**Figure 11:**
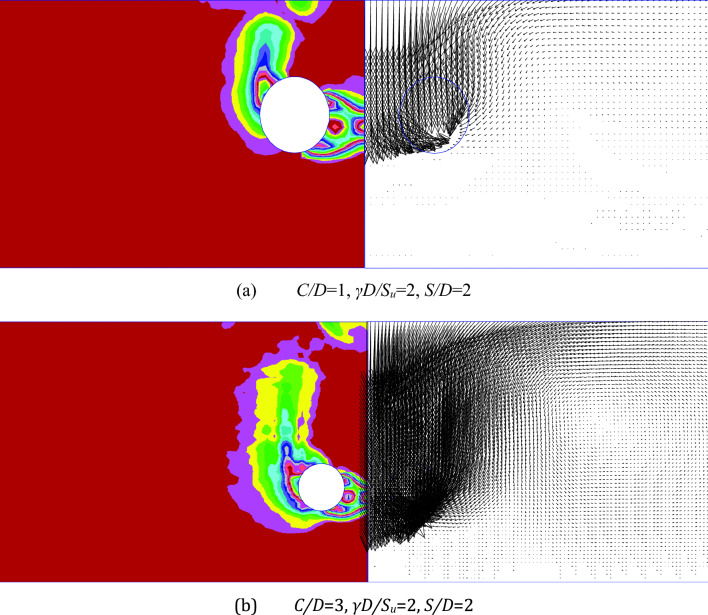
Showcasing the shear strain rate (SSR) plot on the left and the velocity vectors on the right, depicting the collapse scenario for two distinct cases: (**a**) *C*/*D* = 1, *γD*/*S*_*u*_ = 2, *S*/*D* = 2; (**b**) *C*/*D* = 3, *γD*/*S*_*u*_ = 2, *S*/*D* = 2.

Figure 11 showcases the shear strain rate (SSR) and velocity vector plots for two specific depth ratios: *C*/*D* = 1 and 3. These plots correspond to fixed values of *γD*/*S*_*u*_ = 2 and *S*/*D* = 2. The SSR plots provide valuable insights into the failure mechanism, with the most pronounced floor heaving observed in the deeper case (e.g., *C*/*D* = 3). When the culverts are positioned closer to each other (*S*/*D* = 2), a significant interaction between the two culverts is evident, indicated by the SSR band connecting them. However, as the spacing ratio increases, this interaction diminishes, and the plots resemble those of a single culvert. It is important to note that certain SSR plots may exhibit discontinuous contours or "spikes," which can be attributed to the weak equilibrium of the model. This issue can be addressed by increasing the number of elements and steps per stage to achieve improved solution convergence.

### Example: determine the critical internal pressure

In order to ensure the stability of two adjacent culverts that will be simultaneously bored with a spacing of 40 m (center-to-center), it is necessary to determine the minimum internal pressure (*σ*_*t*_) required. These culverts have a diameter (*D*) of 6.0 m and are buried at a depth (*C*) of 18 m in an undrained clayey material with the following properties: undrained shear strength (*S*_u_) = 27 kPa, effective friction angle (*φ*_*u*_) = 0°, and unit weight (*γ*) = 18 kN/m^3^. The site is assumed to be a Greenfield, with no consideration for surface pressure (*σ*_*s*_ = 0). To calculate the absolute minimum internal pressure, the following dimensionless ratios need to be determined: *C*/*D* = 3, *γD*/*S*_*u*_ = 4, and *S*/*D* = 6.67.

By examining the data presented in Fig. 8, it is evident that the critical pressure ratio (PR) for this particular scenario is approximately − 9.2. Rearranging Eq. (2) allows us to calculate the minimum internal pressure (*σ*_*t*_) required to prevent collapse, which amounts to 248.4 kPa. These calculations serve as a valuable design tool for engineers and constructors, enabling them to ensure the stability of the culverts throughout the construction process, particularly in situations where the spacing between the culverts is either relatively large or small.

## Settlement analysis

### Background

The ability to predict ground settlement during construction is crucial, especially in complex projects like twin Culverts where interaction between the structures can occur. While empirical relationships have been used to analyze ground settlement in the past, predicting settlement accurately is still critical in real conditions. O'Reilly and New^34^ proposed a simple method for predicting ground settlement using the double Gaussian equation and the superposition principle, taking into account the interaction between twin culverts. Studies have indicated notable disparities in lining forces between the initial and subsequent culverts, underscoring the importance of considering twin culvert interaction during the design phase.

Studies have been conducted to correlate volume loss to expected ground settlement, including Peck^2^, Veruijt^35^, and numerical researchers such as Addenbrooke and Potts^36^ and Suwansawat and Einstein^37^. Chapman et al.^38^ and Ocak^39^ have proposed modification factors to address this issue. Several studies have explored the settlement of twin culverts through comparisons with numerical methods. For example, Ercelbi et al.^40^, Chen et al.^41^, Divall and Goodey^42^, and Chakeri et al.^43^ have conducted research in this area.

However, there is a lack of criteria for distinguishing twin culvert-induced settlement modes. This study aims to investigate numerical models for ground relaxation and soil movement during Culvert construction, taking into account parameters like depth ratio, soil strength ratio, and Culvert spacing ratio. A practical example is provided to help practitioners understand the process.

### Problem definition and settlement simulations

The problem of predicting ground surface settlement caused by culvert construction is challenging due to various factors such as culvert geometry, material properties, and excavation operations. The complexity increases even more when dealing with twin culvert activity due to the interaction between the two culverts. However, with the advent of digital computers, numerical modelling has become a dominant technique for geotechnical design. The Finite Difference Method (FDM) is one such technique commonly used for geotechnical problems. In this study, the FDM is utilized with a pressure relaxation method developed by Shiau and Sams^32^ for both Culvert stability and settlement problems. The FISH program language of FLAC is extended to facilitate the study of twin culverts in this research. By exploring a wide range of parameters such as Culvert spacing ratio, depth ratio, and soil strength ratio, this study aims to provide practical solutions for practitioners to accurately predict ground relaxation and soil movement during Culvert construction by a tunnel-boring machine.

The utilization of dimensionless ratios in this investigation, such as the depth ratio (*C*/*D*), soil strength ratio (*γD*/*S*_*u*_), and culvert spacing ratio (*S*/*D*), is a customary practice in geotechnical engineering. These ratios serve to simplify the analysis and enhance comprehension of intricate problems. The depth ratio signifies the relationship between the culvert's depth and its diameter, while the soil strength ratio establishes a connection between the effective overburden stress and the undrained shear strength of the soil. The culvert spacing ratio denotes the proportion of the distance between the centers of the culverts to the culvert's diameter. By employing these ratios, a more comprehensive understanding of the interplay among various parameters and their impact on the overall settlement can be achieved.

It is important to note that the assumptions made in this study, such as the use of the Tresca model for the soil and the zero-surcharge assumption, may not always reflect real-world conditions accurately. However, these assumptions have been made for the sake of simplicity and to allow for a more focused analysis of the twin culvert settlement problem. The use of a homogenous clay model also simplifies the analysis, but the results can still provide useful insights into the settlement behavior of twin culverts.

Figure 2 illustrates a representative finite difference mesh employed in the settlement problem analysis. The accurate representation of the entire soil mass through a finite mesh relies on the significance of the boundary conditions, as depicted in Fig. 2. The size of the ground domain for each case was meticulously determined to ensure that the failure zone of the soil body falls within the domain boundaries.

Once the boundary conditions, soil properties, and culvert geometry were established, the internal supporting pressure was gradually reduced from the initial at-rest condition using the developed model. This reduction occurred in incremental steps of 1%. At each step, the stability number was computed, and the surface settlement data was recorded. To simulate the behavior of the ground in the presence of a culvert, the internal pressure (*σ*_*t*_) was gradually decreased using a reduction factor based on the number of relaxation steps. As the pressure decreased, the soil progressively filled the voids within the culvert until the internal forces in the soil achieved equilibrium. This led to a stable circular culvert where no further movement occurred, indicating a state of elastic equilibrium.

However, if the internal pressure reached a level where the internal forces could no longer retain the earth pressures, the nodal forces became unbalanced, resulting in an unstable culvert. It is important to note that this relaxation method involves discrete steps, which may slightly overestimate the stability number at collapse, as the reduction in internal pressure is not continuous. Nevertheless, previous study Shiau and Sams^32^ has demonstrated that reducing the interval size for relaxation significantly improves the accuracy of the analysis.

The analysis revealed that the failure point occurs when the unbalanced forces fail to reach a state of equilibrium during a particular relaxation stage. This critical juncture, known as the collapse stage, can be readily identified by monitoring the history of unbalanced forces. The sudden emergence of this point can also be detected by examining plasticity indicators and velocity plots.

To conduct parametric studies on both stability and settlement responses, this process was implemented using a FISH language, which was employed consistently throughout the research paper. It should be emphasized that all numerical results are presented in dimensionless parameters, rendering their specific values inconsequential.

### Three settlement profiles

In the study, different settlement profiles were observed depending on the spacing ratio (*S*/*D*), with merged profiles resembling a single culvert profile at lower *S*/*D* and partially merged profiles at higher *S*/*D*. Profiles that appeared as two separate culverts were identified if the spacing was more than eight times the inflection point parameter. To analyze the different settlement profiles, the study used three different methods: Method 1, which used the common Gaussian equation from Martos^44^ and Peck^2^ to estimate surface settlements; Method 2, which followed superimposed Gaussian equations from New and O'Reilly^45^ with the additional variable of "*S*" representing the centre-to-centre spacing between Culverts; and Method 3, which analyzed the profiles as two separate Culverts, as demonstrated in studies such as Shiau and Sams^32^. The study found that Method 1 was suitable for merged profiles, while Method 2 was applicable for partially merged profiles. Method 3 was used for profiles appearing as two separate culverts.

3 $$S_{x} = S_{\max } e^{{ - \frac{{x^{2} }}{{2i_{x}^{2} }}}}$$

4 $$V_{s} = \sqrt {2\pi } \times i_{x} \times S_{\max }$$

5 $$S_{x} = S_{\max } \left( {e^{{ - \frac{{\left( {x - 0.5s} \right)^{2} }}{{2i_{x}^{2} }}}} + e^{{ - \frac{{\left( {x + 0.5s} \right)^{2} }}{{2i_{x}^{2} }}}} } \right)$$

**Figure 12:**
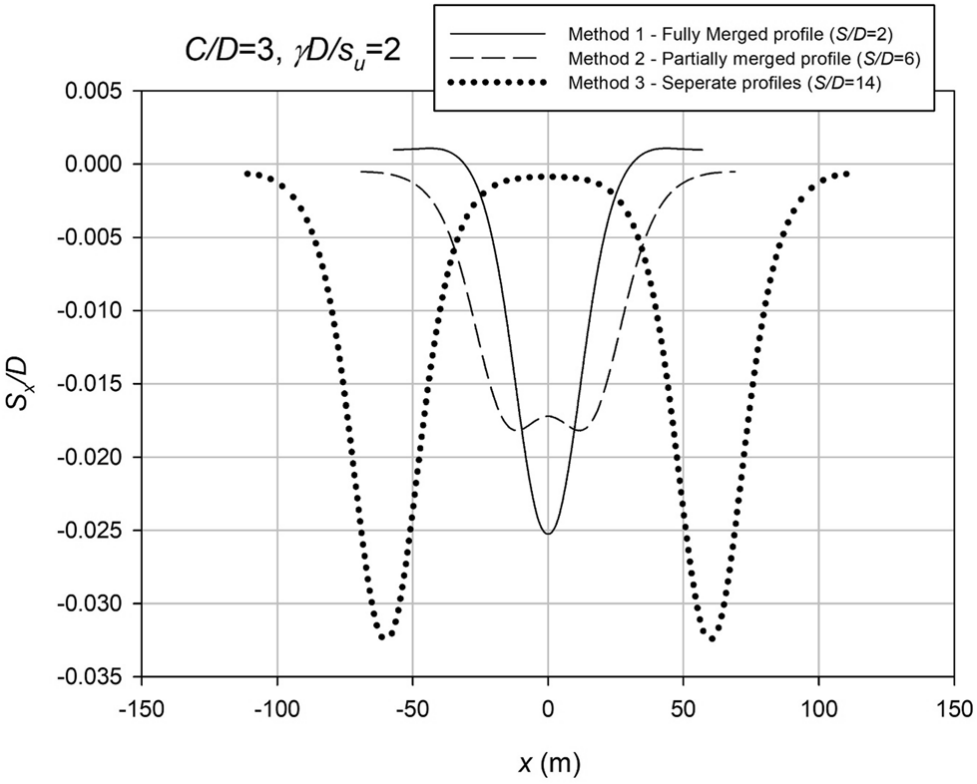
Showcases the three distinct categories employed in this investigation.

The settlement analysis of the fully merged culvert (Method 1) and partially merged culvert (Method 2) involves the use of regression analysis to fit mathematical equations to the collected data. Equation (3) accurately represents the settlement profiles for Method 1, while Eq. (4) captures the settlement profiles for Method 2. Figure 12 provides a visual representation of these methods, demonstrating their distinct characteristics.

To perform the regression analysis, MATLAB and the curve fitting toolbox were utilized. The data collected from both the collapse and working condition stages were employed in this analysis. The goal was to find the best-fit parameters for the equations by adjusting the trough width parameter (*i*_*x*_) while keeping the maximum settlement parameter (*S*_max_) constant at the observed maximum settlement value.

To ensure the accuracy and reliability of the regression models, a robust bi-square regression technique combined with a trust-region algorithm was employed. This approach accounted for potential outliers and variations in the data, resulting in precise and robust models. The determination coefficient (*R*^2^) values, which indicate the goodness of fit between the models and the data, exceeded 0.97 for all cases, demonstrating a strong correlation.

The high *R*^2^ values indicate the models' ability to accurately capture the settlement behavior for both Method 1 and Method 2. These regression models provide a practical and reliable tool for settlement analysis in twin culvert systems, allowing engineers to estimate settlements based on the specific parameters of each method.

**Figure 13:**
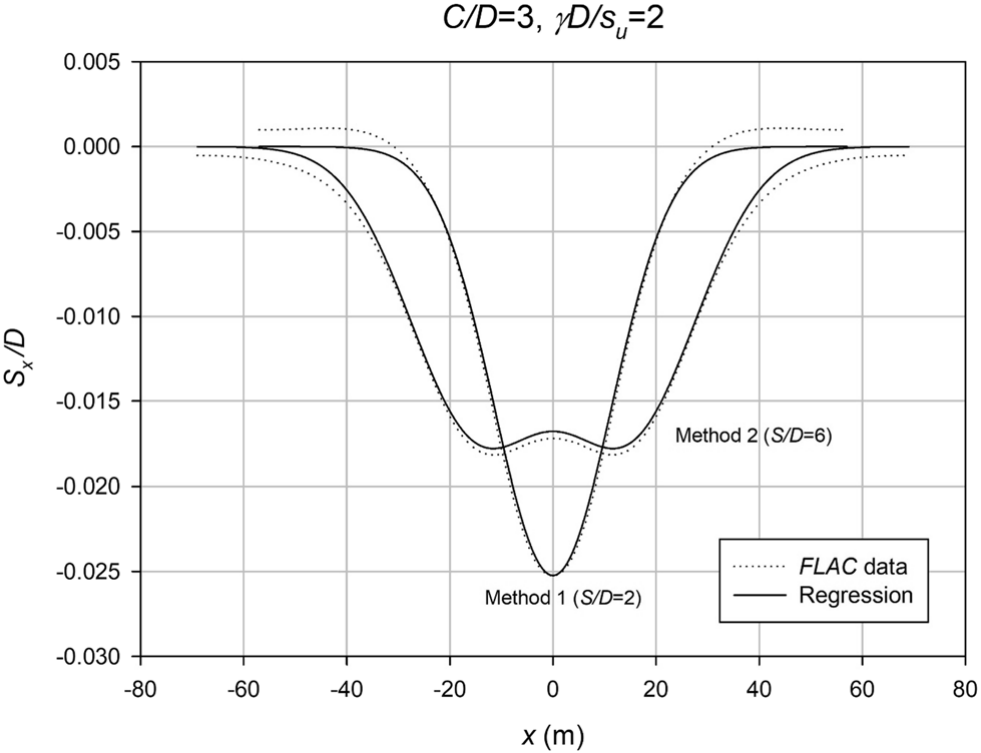
Comparison of FLAC results with equation regression.

Figure 13 provides comprehensive curve fitting examples for both Methods 1 and 2, with a specific focus on *S*/*D* ratios of 2 and 6 (*C*/*D* = 3, *γD*/*S*_*u*_ = 2). The curve fitting process was conducted using MATLAB and the curve fitting toolbox, allowing for accurate regression analysis of the settlement data collected at both the collapse and working condition stages. The resulting *i*_*x*_ values, which represent the inflection point parameter, were obtained through this robust methodology and have consistently proven to be reliable for each respective case. These findings highlight the effectiveness and practicality of the curve fitting approach in estimating *i*_*x*_ values for different scenarios in settlement analysis.

**Figure 14:**
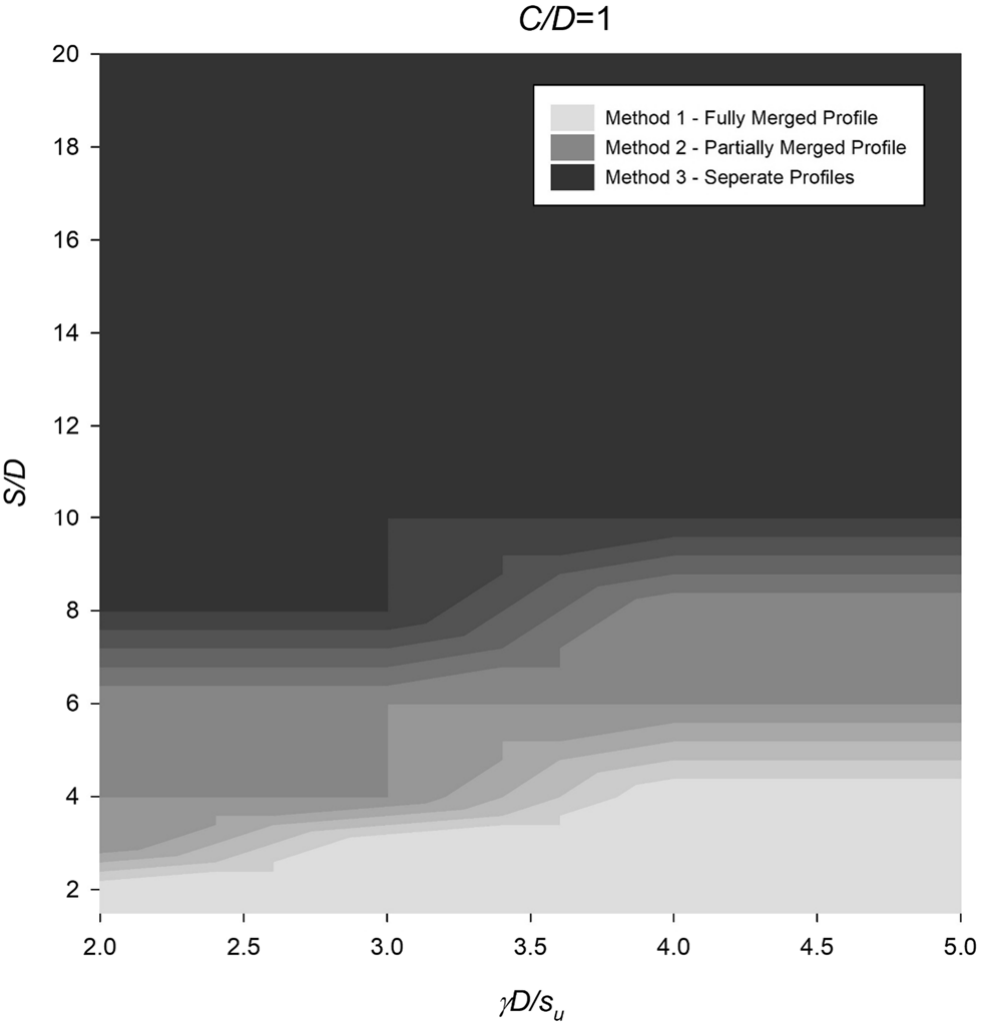
Contour plot showcasing the recommended method of settlement analysis for the case of *C*/*D* = 1.

**Figure 15:**
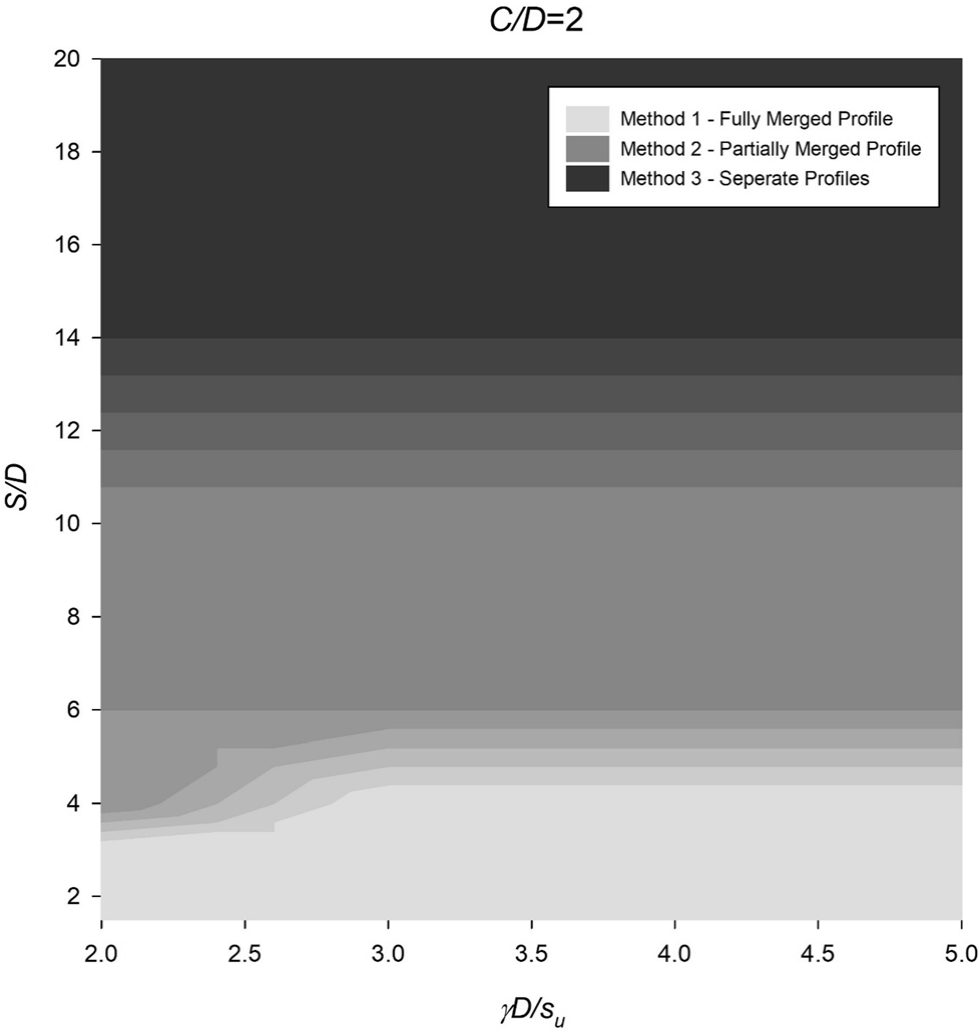
Contour plot illustrating the recommended method of settlement analysis for the case of *C*/*D* = 2.

**Figure 16:**
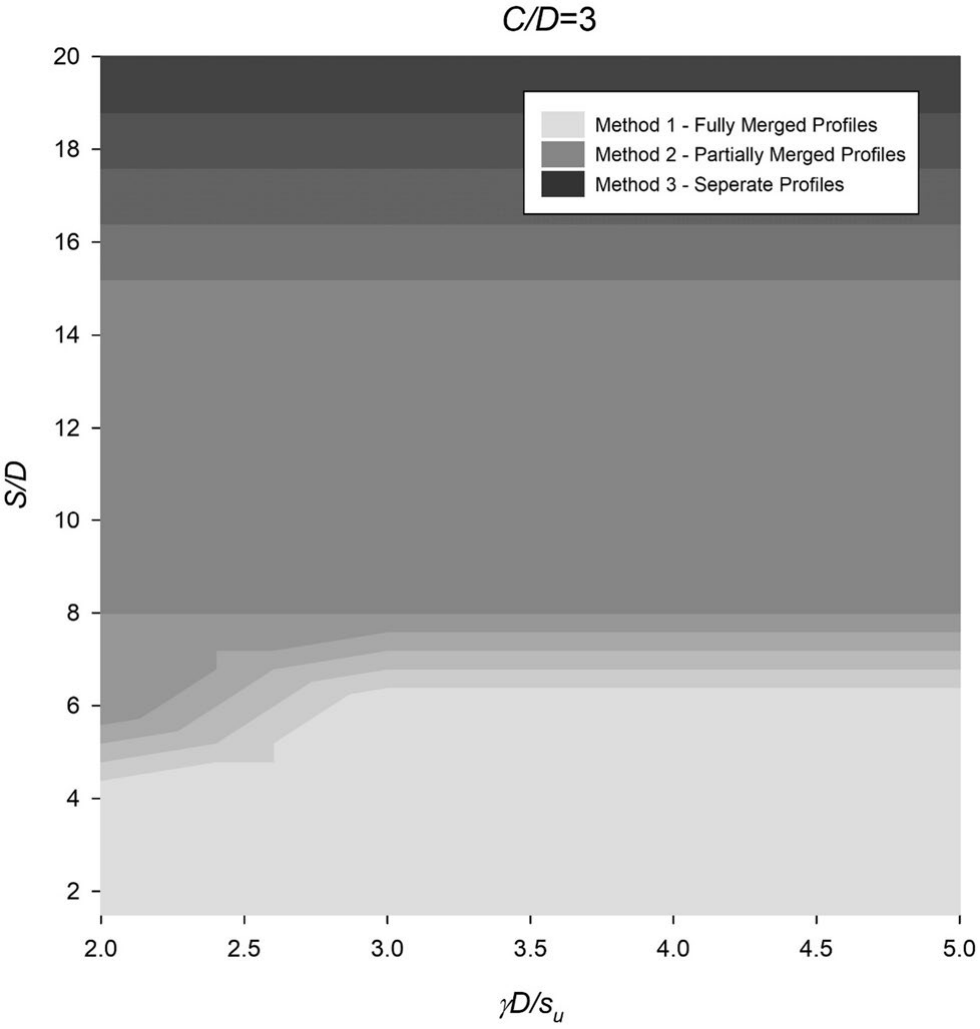
Contour plot illustrating the recommended method of settlement analysis for the case of *C*/*D* = 3.

**Figure 17:**
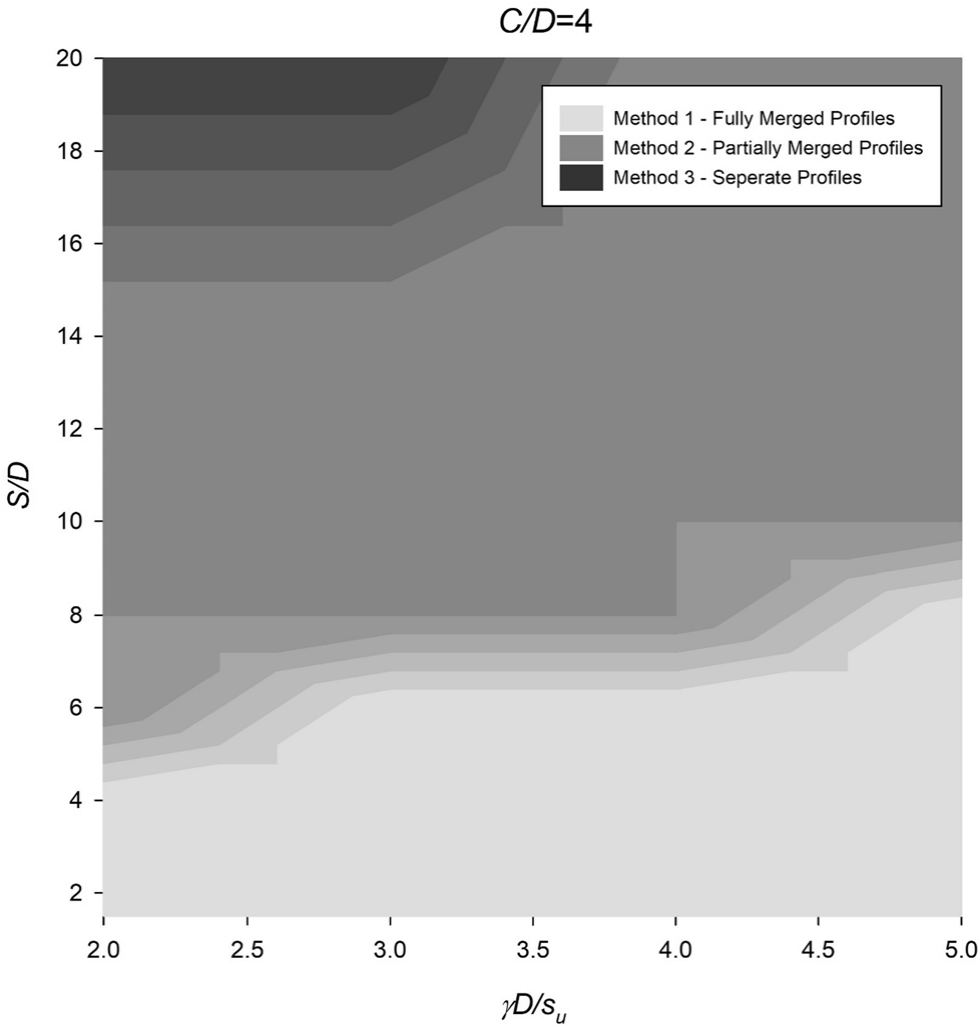
Contour plot illustrating the recommended method of settlement analysis for the case of *C*/*D* = 4.

**Figure 18:**
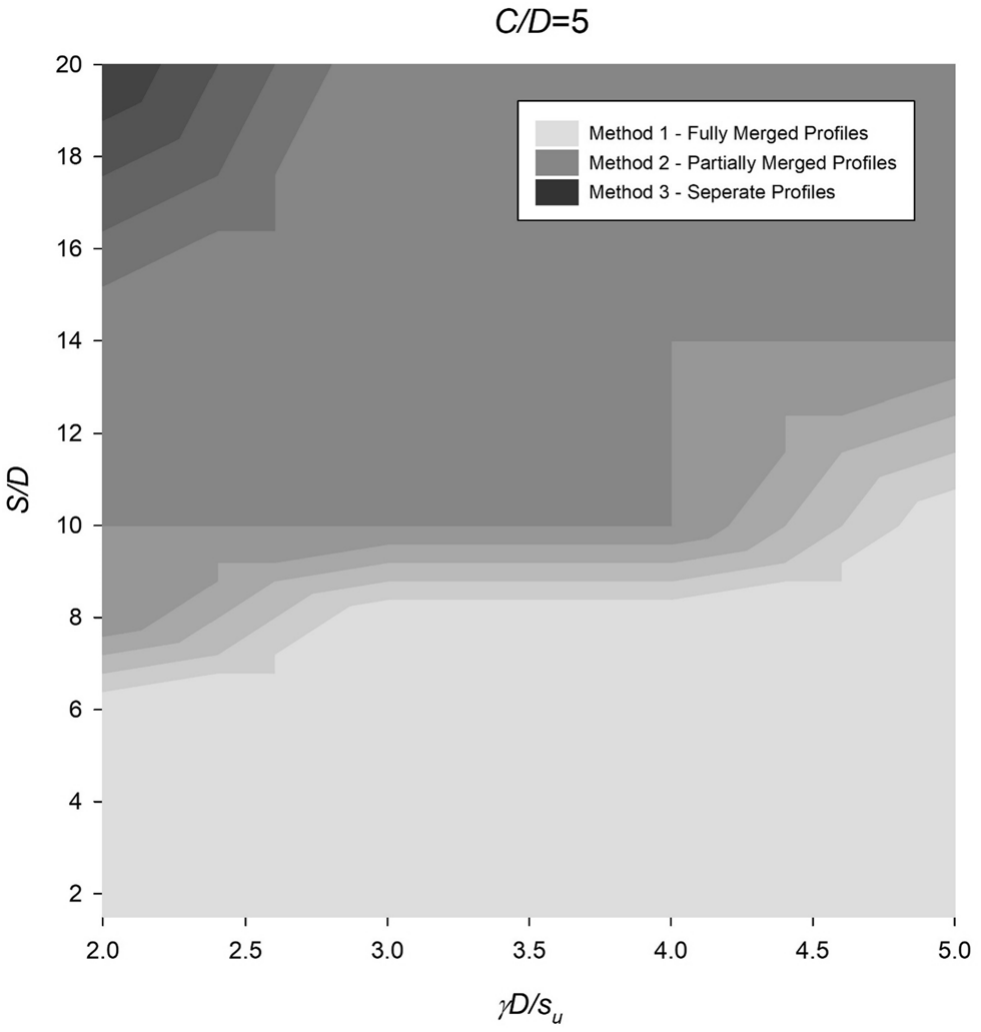
Contour plot illustrating the recommended method of settlement analysis for the case of *C*/*D* = 5.

However, the settlement profile for twin culverts can vary significantly depending on the spacing ratio, with fully merged, partially merged, or separate culverts being observed under different conditions. To address this, Figs. 14, 15, 16, 17 and 18 provide a chart to estimate which method is required based on depth, soil strength, and spacing ratios. Notably, stronger soils tend to require Method 2 at lower *S*/*D* ratios, while deeper depths delay the transition from Method 2 to Method 3 until larger spacing ratios are reached.

### Discussing settlement results

This section aims to present and analyze the settlement results of twin culverts at the collapse stage, with subsections dedicated to each settlement profile method.

#### Method 1: fully merged profile

**Figure 19:**
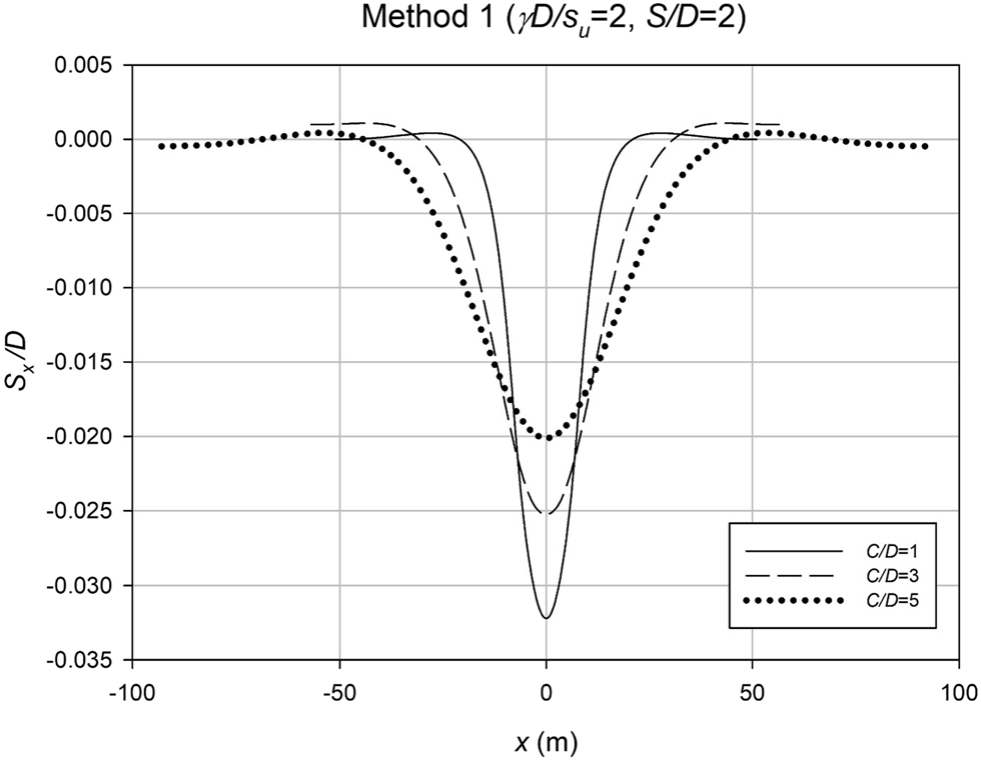
Variation of settlement profiles for a representative case using Method 1, depicting the impact of the depth ratio (*C*/*D*) on settlement behavior.

Figure 19 displays settlement profiles obtained using Method 1, where the depth ratio (*C*/*D*) is varied while keeping the strength ratio (*γD*/*S*_*u*_) and spacing ratio (*S*/*D*) constant. The profiles clearly reveal an interesting trend: as the depth ratio increases, the settlement trough becomes wider and shallower. This is in stark contrast to the shallow case, where a narrow but deep trough is observed, indicating different settlement patterns associated with varying depth ratios.

**Figure 20:**
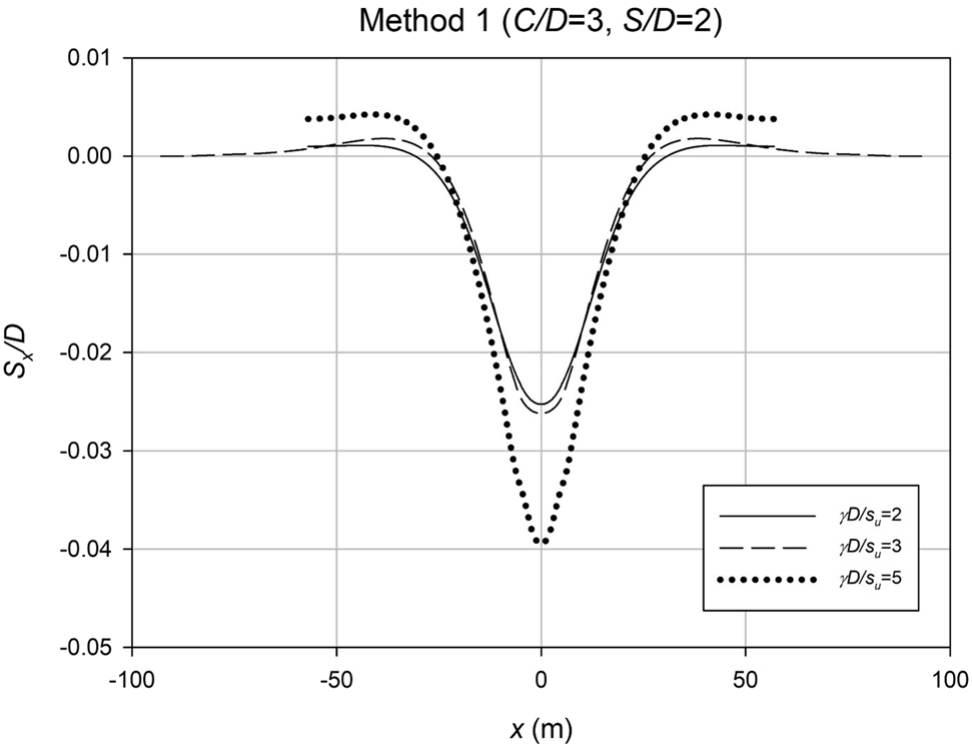
Settlement profiles for a representative case using Method 1, illustrating the influence of the strength ratio (*γD*/*S*_*u*_) on settlement behavior.

In Fig. 20, settlement profiles are presented for a fixed spacing ratio (*S*/*D*) and depth ratio (*C*/*D*), but with different strength ratios (*γD*/*S*_*u*_). The chart effectively demonstrates that the case involving weaker soil, characterized by a lower *γD*/*S*_*u*_ ratio (*γD*/*S*_*u*_ = 5), exhibits a significantly larger maximum settlement at collapse. This observation aligns with the expected behavior, as weaker soils are anticipated to undergo greater movement during a collapse event.

These findings from Figs. 19 and 20 provide valuable insights into the settlement behavior of the studied culvert system under different design parameters. The results emphasize the significance of considering depth ratio, strength ratio, and spacing ratio in culvert design to optimize stability and minimize settlement.

**Figure 21:**
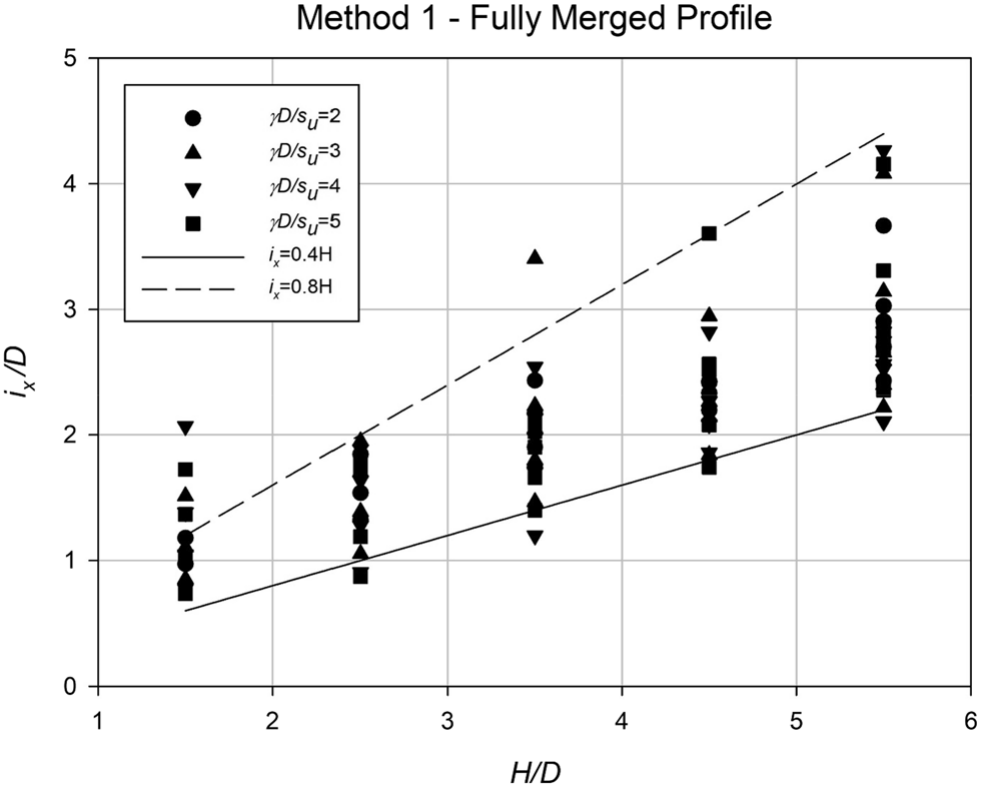
Relationship between the dimensionless parameter *i*_*x*_/*D* and *H*/*D* for all data obtained using Method 1, with varying values of the strength ratio (*γD*/*S*_*u*_).

**Figure 22:**
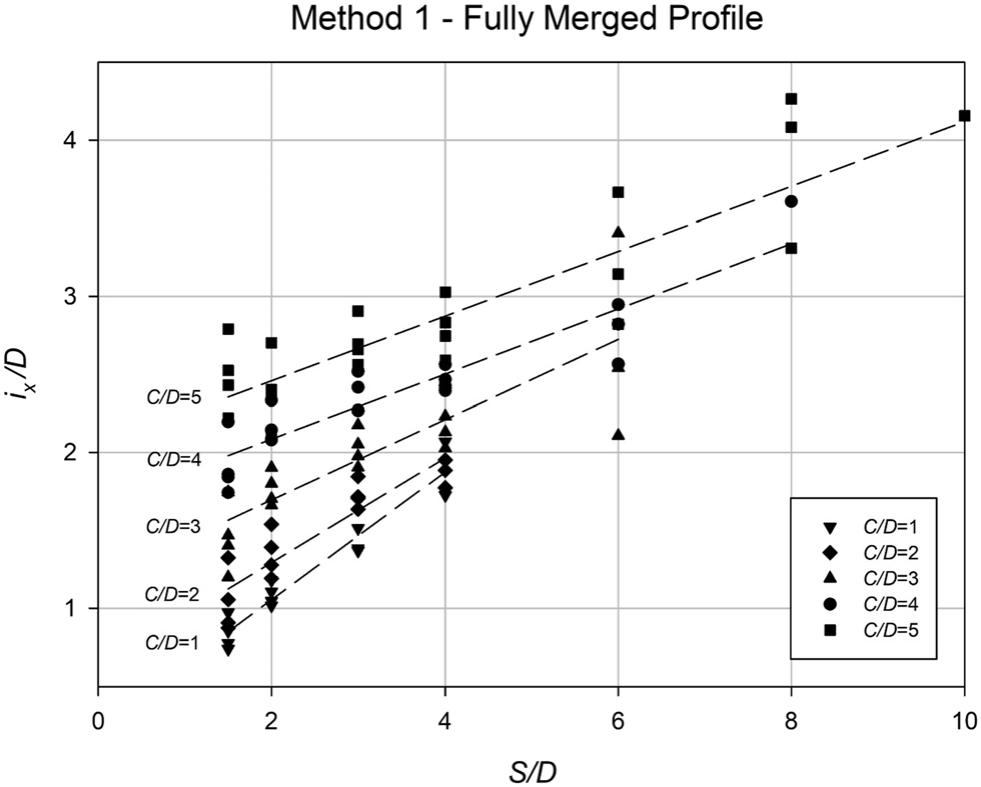
Relationship between the dimensionless parameter *i*_*x*_/*D* and *S*/*D* for all data obtained using Method 1, with varying values of the depth ratio (*C*/*D*).

A regression approach has been utilized to analyze the settlement data and determine the values of *i*_*x*_ for each scenario. In order to make the analysis more practical, *i*_*x*_ has been normalized by dividing it by the diameter *D*. The analysis of these normalized *i*_*x*_ values is presented in Figs. 21 and 22, which highlight the dependence of *i*_*x*_/*D* on both the depth ratio (*H*/*D*) and the spacing ratio (*S*/*D*). Figure 21 illustrates the relationship between *i*_*x*_/*D* and *H*/*D*, indicating a linear correlation where the data points fall within the range of approximately 0.4H to 0.8H. Conversely, no significant relationship can be observed between *i*_*x*_/*D* and *γD*/*S*_*u*_, as the data points appear scattered. However, Fig. 22 clearly demonstrates the relationship between *i*_*x*_/*D* and the spacing ratio *S*/*D* for various *C*/*D* ratios. The steepest gradient in this relationship is observed in shallower cases. Based on the information presented in the figure, Eq. (6) has been derived to estimate *i*_*x*_/*D* at collapse for any twin culvert scenario modelled using "Method 1," taking into account the values of *C*/*D* and *S*/*D*.

6 $$\frac{{i}_{x}}{D}=\left(\frac{C}{D}\right)\left[-0.05\left(\frac{S}{D}\right)+0.47\right]+0.44\left(\frac{S}{D}\right)-0.25$$

#### Method 2: partially merged profiles

**Figure 23:**
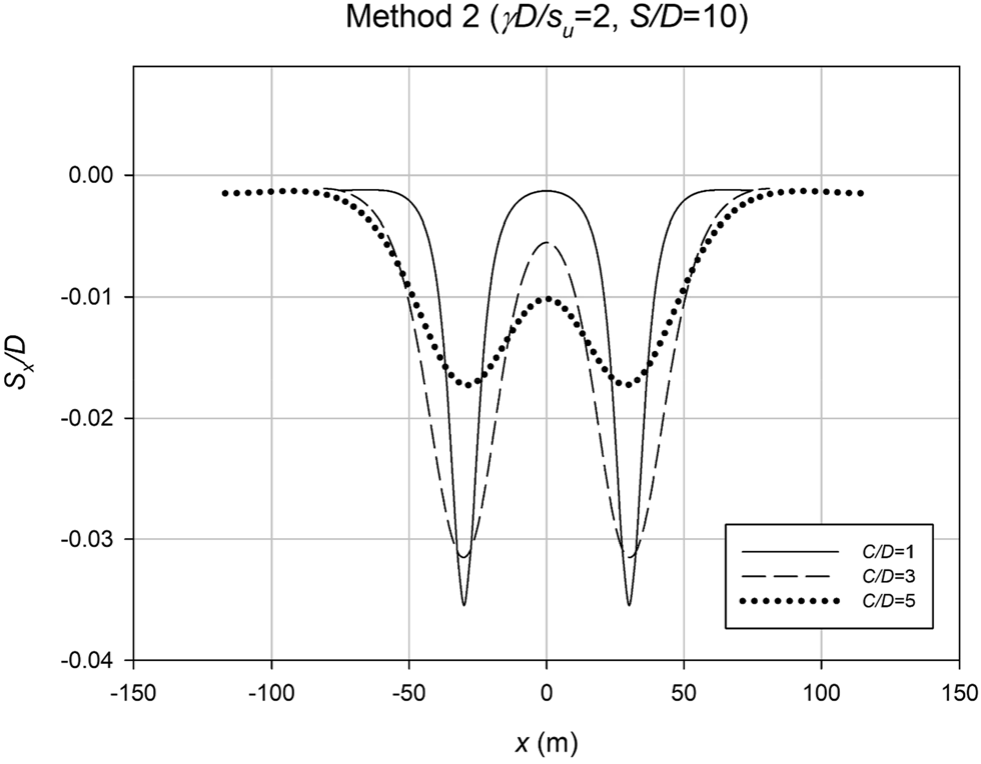
Settlement profiles for a typical Method 2 case with varying depth ratio (*C*/*D*).

**Figure 24:**
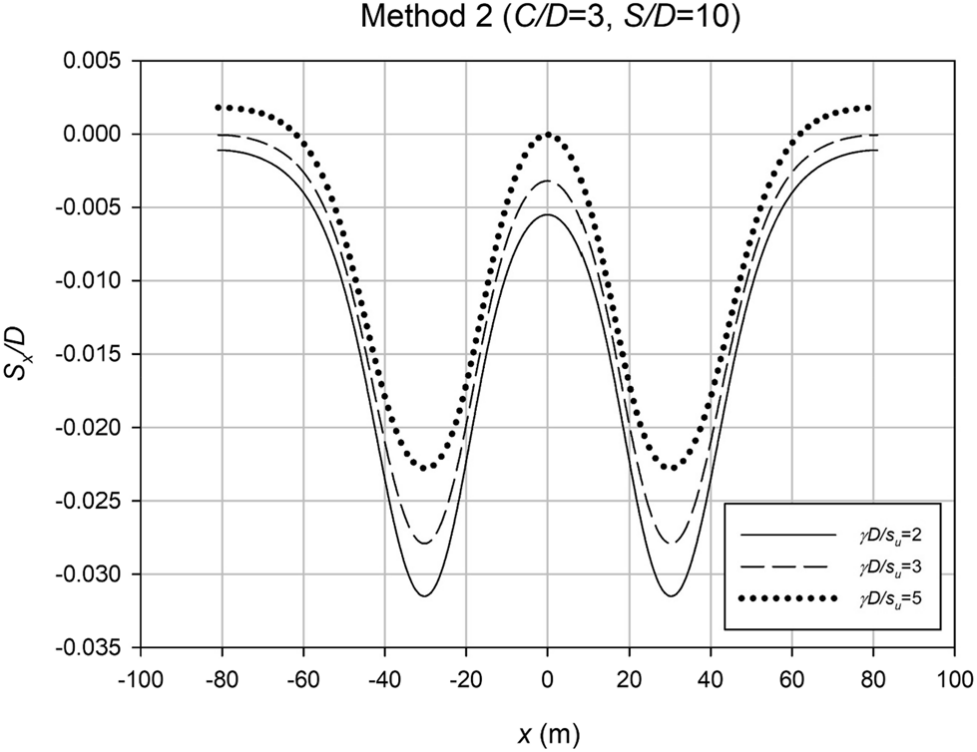
Settlement profiles for a typical Method 2 case with varying strength ratio (*γD*/*S*_*u*_).

The settlement profiles were examined for Method 2 to investigate the impact of varying the depth ratio (*C*/*D*), while maintaining the strength ratio (*γD*/*S*_*u*_) and spacing ratio (*S*/*D*) constant. Figure 23 displays these profiles, revealing wider settlement patterns in deeper scenarios, with smaller maximum settlement compared to shallower cases, aligning with expectations. In contrast, Fig. 24 presents settlement profiles for cases with fixed *C*/*D* and *S*/*D* values but varying *γD*/*S*_*u*_. The results indicate that weaker cases exhibit lower maximum settlement compared to stronger cases, likely due to increased interaction effects. This phenomenon allows the stronger cases to undergo further relaxation before reaching failure.

**Figure 25:**
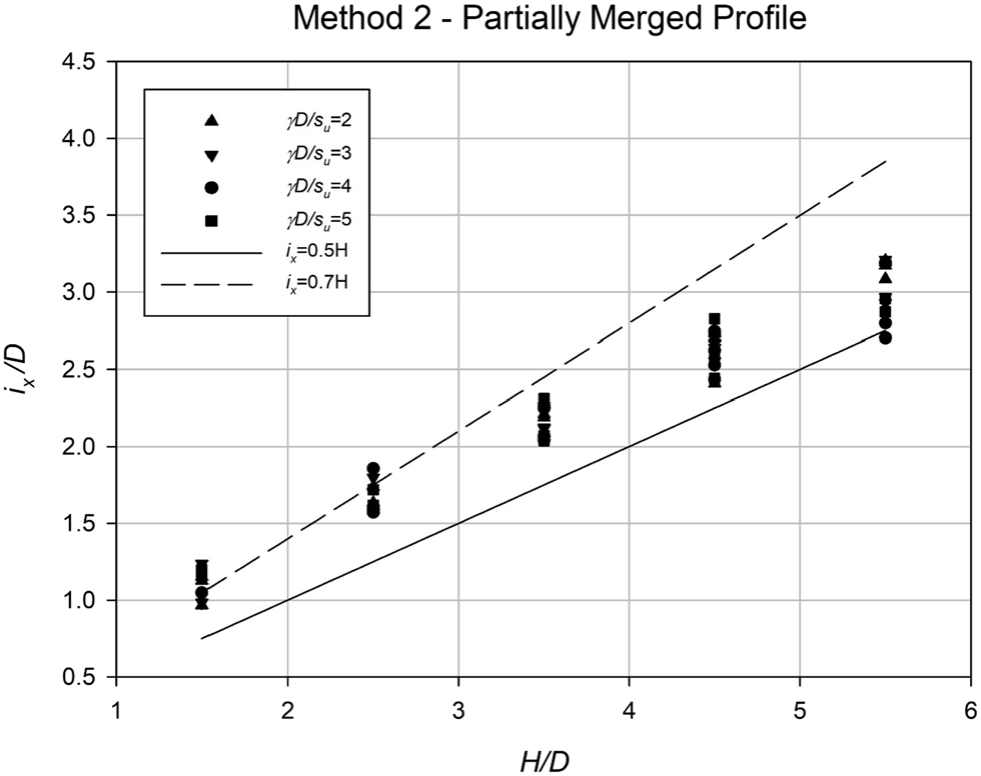
Normalized trough width parameter (*i*_*x*_/*D*) plotted against the depth ratio (*H*/*D*) for all method 2 data, with varying strength ratio (*γD*/*S*_*u*_).

**Figure 26:**
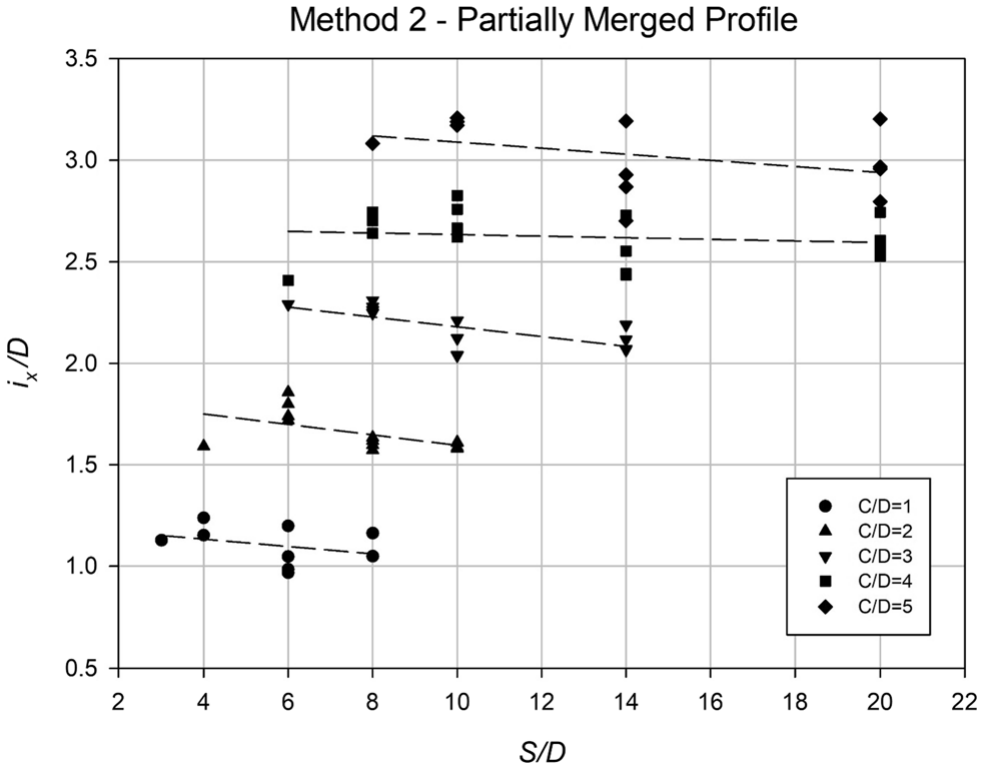
Normalized trough width parameter (*i*_*x*_/*D*) plotted against the spacing ratio (*S*/*D*) for all method 2 data, with varying depth ratio (*C*/*D*).

The settlement data obtained from the profiles in Method 2 were analyzed using regression analysis to determine an accurate *i*_*x*_ value for each case. Similar to Method 1, the *i*_*x*_ value was normalized with Culvert diameter, *D*, to make the results dimensionless. Figures 25 and 26 show some analysis of these *i*_*x*_ results. Figure 25 displays the correlation between *i*_*x*_/*D* and *H*/*D* for various *γD*/*S*_*u*_ values. The results reveal a linear association between *i*_*x*_/*D* and *H*/*D*, indicating that O’Reilly and New’s correlation remains valid in practice. Compared to the graph in Method 1, Fig. 21, the new data is more precise, with a k range of roughly 0.5–0.7.

Figure 26 depicts the relationship between the normalized trough width parameter (*i*_*x*_/*D*) and the spacing ratio (*S*/*D*) for various depth ratio (*C*/*D*) values. The analysis reveals that the depth ratio (*C*/*D*) has a notable influence on *i*_*x*_/*D*, while the spacing ratio (*S*/*D*) has a relatively minor impact, indicated by the nearly horizontal alignment of the data points and regression lines. These findings led to the development of Eq. (7), which provides an approximate estimation of *i*_*x*_/*D* for any given *C*/*D* value.

7 $$\frac{{i}_{x}}{D}=0.49\left(\frac{C}{D}\right)+0.81$$

#### Method 3: separate profiles

According to Shiau and Sams^32^, it has been observed that in cases where twin culverts are positioned at a significant distance from each other, their settlement behavior is relatively independent. Consequently, the settlement analysis of such twin culverts can be effectively performed by considering each culvert as a separate entity. The rationale behind this approach is that the spatial separation between the culverts minimizes their mutual influence, thereby allowing their settlement profiles to be analyzed individually. This simplification greatly facilitates the settlement analysis process and provides a practical solution for engineering design and assessment of twin culvert systems.

### Example: prediction of settlement of twin culverts

In the construction plan, twin culverts with a diameter of 5 m and a cover depth of 20 m are being simultaneously bored. The spacing between the culverts is 20 m. Key soil properties, including a unit weight of 18 kN/m^3^ and an undrained shear strength of 27 kPa, have been obtained through preliminary soil lab tests. However, accurately predicting the resulting settlement profile poses a challenge due to the contractors' requirement of a maximum volume loss limit of 2% per culvert.

To address this challenge, it is crucial to employ an appropriate equation that can provide an accurate estimation of the settlement profile. By selecting a suitable equation and considering the given soil properties and culvert dimensions, the project team can make informed decisions and ensure that the anticipated settlement remains within the allowable limits. Careful consideration and analysis are essential to meet the contractors' requirements and ensure the successful completion of the twin culvert construction project.

To estimate the settlement profile, several dimensionless ratios have been calculated: *C*/*D* = 4, *γD*/*S*_*u*_ = 3.33, and *S*/*D* = 4. The analysis conducted in Fig. 17 leads to the conclusion that "Method 1—Fully Merged Profiles" along with the standard single Gaussian equation are suitable for evaluating these ratios. By referring to the data presented in Fig. 21 or Eq. (6), an estimation of *i*_*x*_/*D* can be made, yielding a value of approximately 1.6. This corresponds to an *i*_*x*_ value of 8 m.

Considering the specified volume loss limit of 2% per culvert, the total contraction area is computed using Eq. (4), resulting in a value of 0.79 m^2^. By rearranging the equation, *S*_max_, the maximum settlement, is determined to be 0.039 m. Finally, Eq. (3) is utilized to establish a settlement profile equation [Eq. (8)], which provides a practical tool for predicting the ground surface settlement profile in this particular scenario.

8 $$S_{x} = 0.039e^{{ - \frac{{x^{2} }}{{2 \times 8^{2} }}}}$$

## Conclusion

The current study has made significant strides in developing a robust numerical procedure that effectively predicts soil relaxation during the construction of twin circular culverts. This innovative approach stands out for its ability to automatically generate finite difference grids and meshes based on geometric inputs, along with capturing settlement output data for each relaxation step. Rigorous comparisons against upper and lower bounds, as well as previous experimental research, have consistently demonstrated a high level of agreement. These results attest to the accuracy and reliability of the pressure relaxation method as a valuable design tool for practical applications in the field.

However, it is essential to acknowledge a limitation in the current study. The use of the Tresca model for soil behavior and the zero-surcharge assumption, while providing a conservative and simplified approach, may not always reflect real-world conditions accurately. Real-world scenarios often involve varying surcharge loads and more complex soil behaviors, which may not be fully captured by these assumptions.

In order to increase the applicability of the research, settlement data from different stages, including the critical collapse stage and three pre-collapse stages, were carefully extracted and analyzed using MATLAB. The data were fitted using either a standard Gaussian or a modified twin-Gaussian curve. The results demonstrated a strong correlation between the settlement patterns predicted by the FLAC model and the Gaussian and Twin-Gaussian curves. This correlation indicates that the proposed approach has significant potential as an initial tool in the industry.

Moreover, through an extensive parametric study, comprehensive design charts have been developed that offer practical examples of culvert geometries and complex soil layers. These design charts serve as valuable references for determining the inflection point parameter *i*_*x*_/*D* in both Method 1 and Method 2 cases. By combining these design charts with the empirical method, engineers and practitioners gain access to a powerful and practical tool for accurately estimating surface settlement in twin culvert systems.

In summary, this study has not only presented an accurate numerical procedure for predicting soil relaxation during the construction of twin circular Culverts, but it has also effectively demonstrated the potential of the empirical method for practical applications in the industry. The results highlight the reliability and practicality of the developed approach, providing engineers with a valuable solution for tackling complex real-world projects with confidence.

## Data Availability

All data, models, or codes that support the findings of this study are available from the corresponding author upon reasonable request.
